# Supplementary material for: Optogenetic control of cellular forces and mechanotransduction
Source: Nat Commun. 2017 Feb 10;8:14396. doi: 10.1038/ncomms14396 (PMC5309899; doi:10.1038/ncomms14396)
Supplement: Supplementary Information — Supplementary Figures [file ncomms14396-s1.pdf]

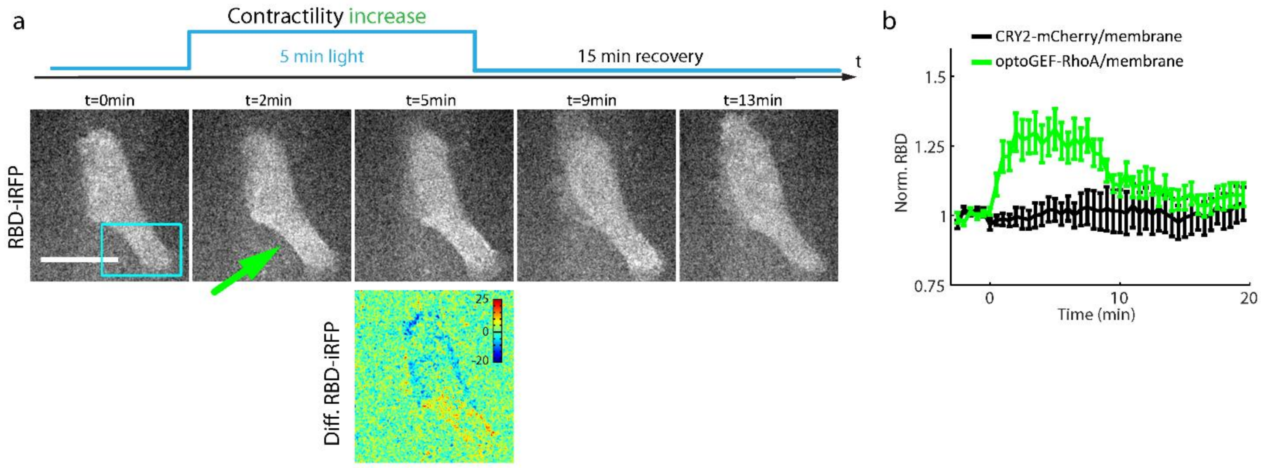

Supplementary Figure 1: Local optogenetic activation of RhoA

(a) iRFP images of MDCK cells expressing optoGEF-RhoA, CIBN-GFP-CAAX and RBD-iRFP before, during and after 5 minutes of local illumination with blue light (top). Difference in fluorescence intensity between t=5min and t=0min (bottom). (b) Quantification of RBD signal over time in the activated area divided by the value in a not activated area and normalized to baseline. In green, cells expressing optoGEF-RhoA, CIBN-GFP-CAAX and RBD-iRFP (n=19); in black, control cells expressing CRY2-mCherry, CIBN-GFP-CAAX and RBD-iRFP (n=12). Error bars are s.e.m. Scale bar is 20 μm.

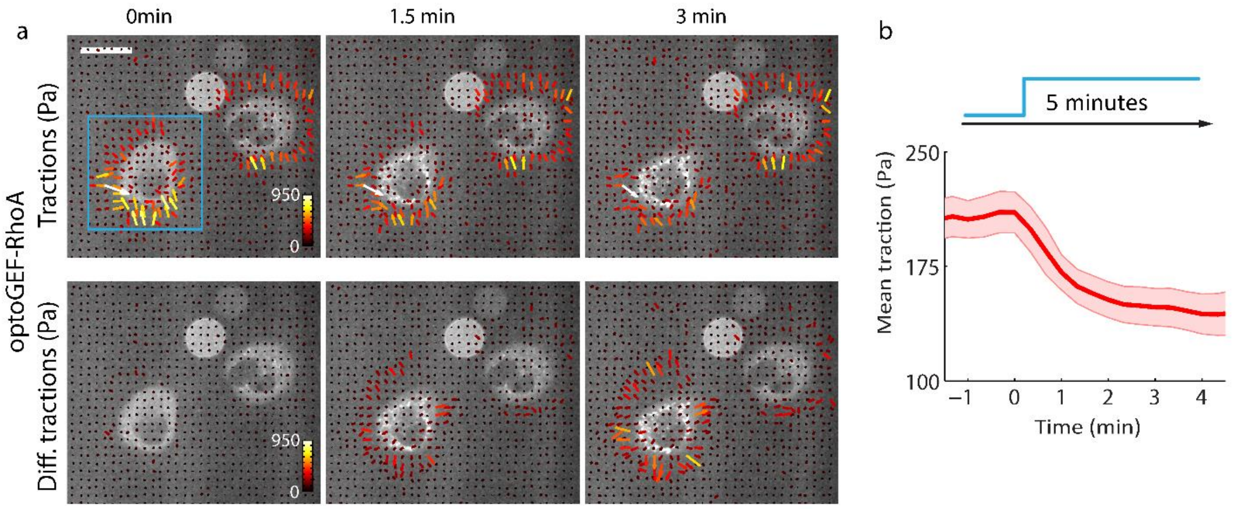

Supplementary Figure 2: Relaxation of contractility in cells stably expressing mito-CIBN-GFP and optoGEF-RhoA

(a) Images of optoGEF-RhoA-mCherry signal just before optogenetic activation for MDCK cells stably expressing optoGEF-RhoA and mito-CIBN-GFP. At time t=0, the cell highlighted by a blue square was illuminated (one pulse of blue light every 20 seconds for 5 minutes). Traction forces (upper images) and differential tractions (with respect to baseline) during optogenetic activation are represented with color-coded arrows. (b) Quantification of the mean traction amplitude over time for activated cells (n=8, shaded area indicates s.d.). Scale bar is 20 μm.

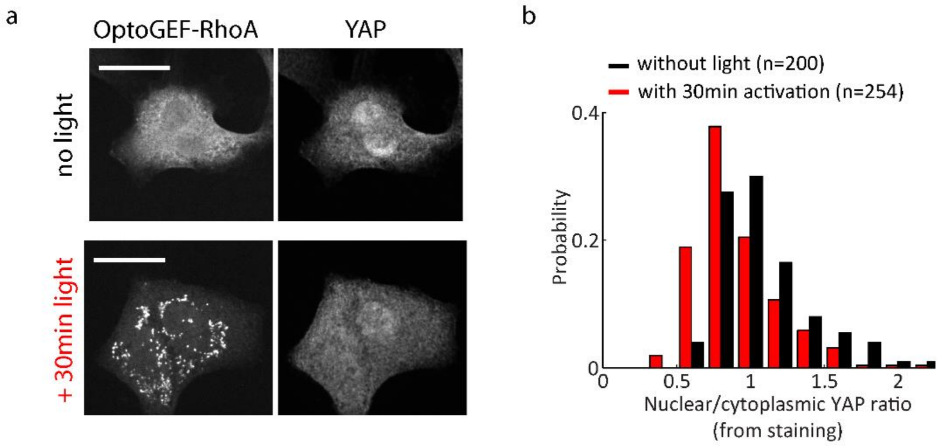

Supplementary Figure 3: Optogenetic relaxation of cell contractility decreases nucleus vs. cytoplasmic YAP ratio.

(a) Representative images of cells stained for YAP. The cells were transfected with mito-CIBN-GFP and optoGEF-RhoA and illuminated (bottom) or not (top) with blue light for 30min before adding PFA. Left, mCherry signal from optoGEF-RhoA; right, far red signal from stained YAP. Scale bars are 20 µm. (b) Distribution of the nuclear/cytoplasmic YAP ratio in the experimental population for single cells on 12kPa polyacrylamide gels obtained from immunostainings. Cells expressing optoGEF-RhoA and mito-CIBN-GFP were either subjected to blue light (red bars, n=254 cells, one pulse every 45 seconds for 30 minutes) or to no light (black bars, n=200 cells). The two populations were significantly different ($p<0.0001$, parametric Wilcoxon-Mann-Whitney test).
